# Identification of a common haplotype in carriers of rob(1;29) in 32 Italian cattle breeds

**DOI:** 10.1038/s41598-023-46341-3

**Published:** 2024-01-24

**Authors:** Matteo Cortellari, Arianna Bionda, Luigi Liotta, Fiorella Sbarra, Pietro Parma, Paola Crepaldi

**Affiliations:** 1 Dipartimento di Scienze Agrarie e Ambientali—Produzione, Territorio, Agroenergia, University of Milan, Via Celoria 2, 20133 Milan, Italy; 2 Dipartimento di Scienze Veterinarie, University of Messina, Viale Palatucci 13, 98168 Messina, Italy; 3 National Association of Italian Beef-Cattle Breeders (ANABIC), 06132 San Martino in Colle, Perugia Italy

**Keywords:** Genetics, Cytogenetics

## Abstract

Robertsonian translocation 1;29 (rob(1;29)), a widespread chromosomal anomaly affecting cattle fertility, appears to have originated from a common ancestor. This study utilizes routine SNP data to investigate the chromosomal region associated with rob(1;29) and confirm the presence of a shared haplotype among carriers in diverse Italian breeds. Three datasets were employed: Dataset 1 included 151 subjects from 5 beef cattle breeds genotyped with the GGP Bovine 33 k SNP chip; Dataset 2 encompassed 800 subjects from 32 Italian breeds genotyped with the Illumina 50 k SNP chip, sourced from the BOVITA dataset; Dataset 3 combined Dataset 2 with 21 karyologically tested subjects from breeds with a high carrier frequency, genotyped using the Affymetrix 65 K SNP chip. FST analysis pinpointed a distinctive genomic region on the first six Mb of BTA29, the centromeric region involved in the translocation. Haplotype comparisons within this non-recombining region revealed a common haplotype shared among all carriers, supporting the theory of a common ancestor. Principal component and haplotype analysis allowed clear differentiation of rob(1;29) homozygous and heterozygous carriers. Expanding to Dataset 2 revealed rob(1;29) carriers in unexpected breeds, all sharing the same ancestral haplotype. Notably, previously untested breeds, including Cinisara, exhibited a high carrier prevalence (nearly 50%), confirmed by karyological analysis. This study validates the presence of a shared haplotype among all identified rob(1;29) carriers, reinforcing the common ancestor theory as the origin of this translocation's spread throughout the cattle population. Furthermore, it underscores the potential of SNP data analysis as a rapid, accurate, and cost-effective tool for broad rob(1;29) screening, given the translocation's consistent nature across all analyzed breeds.

## Introduction

Reproductive efficiency is one of the key criteria for achieving proper genetic progress and financial advantages in cow breeding. Chromosomal abnormalities are one of the factors that can jeopardize this aspect. The discovery of the Robertsonian translocation 1;29 (rob(1;29)) in this species^1^ and the unequivocal confirmation of its detrimental effects on the fertility of the carrier subjects^2–6^ marked a turning point in the advancement of reproductive effectiveness.

Robertsonian translocations, also known as centric fusions, are characterized by the lack of genetic material loss or gain since they result from the fusion of two acrocentric chromosomes across their respective centromeres. The decline in reproductive efficiency in carrier subjects is caused by the generation of genetically imbalanced gametes, which results in the formation of unbalanced embryos that can only survive until the eighth day of gestation^7,8^. As a result, the calving interval is lengthened, and a new fertilization is required.

Later analyses have revealed a widespread occurrence of this abnormality and a definite majority presence in breeds intended for meat production^9^. The explanation for this preference for meat-producing breeds, as well as its origin, are still being disputed.

Because of the evident impact of this aberration on reproductive efficiency, many European countries have initiated early screening programs to eliminate carrier subjects from reproduction^10^ and Italy is one of these countries with the highest level of commitment, with a total of more than 22,000 subjects examined since 1976 (Parma P, personal communication).

Along with these screening procedures, the advancement of cytogenetics applied to the bovine species has allowed researchers to demonstrate how rob(1;29) has a different genomic structure than what is predicted by the rob creation mechanism. First evidence emerged by examining synaptonemal complexes, which revealed an imperfect pairing during the meiosis process, in contrast to the perfect matching expected between BTA1, BTA29, and rob(1;29)^11^. Following that, a discordant mapping of a cosmid between BTA29 and rob(1;29) was highlighted in a physical mapping work in the bovine genome^12^, a situation confirmed in a subsequent investigation^13^. According to current knowledge, after the fusion of BTA1 and BTA29, the centromeric region of BTA29, around 5.4 Mb, inverted and migrated onto the q arm of rob(1;29)^14^. The absence of meiotic recombination in this 5.4 Mb genomic region is the primary effect of this peculiar genomic structure, making this region extremely stable from a genetic point of view. Despite the identification of thousands of carriers, none has ever been recognized as de novo. Thus, several authors^9,10,14^ suggested that the origin of rob(1;29) translocation in all bovine specimens traces back to a single ancestor. Should this hypothesis hold true, it is plausible that the implicated region has preserved an ancestral haplotype, which has since become disseminated among all carriers of this translocation.

In light of this, the present study analyses the SNP data routinely collected and analyzed by breed associations to evaluate the possibility of identifying a region differentiating carriers and clear subjects and to verify the existence of a conserved haplotype among carrier animals spanning 32 distinct Italian cattle breeds of diverse origins.

## Results and discussion

### Dataset 1

As shown in Table 1, Dataset 1 included 151 cytogenetically tested animals belonging to five Italian beef cattle breeds managed by the National Association of Italian Beef-Cattle Breeders (ANABIC) (i.e., Maremmana, Romagnola, Chianina, Podolica, and Marchigiana), which routinely undergo karyotypic tests aimed at the identification of chromosome translocations. Genomic data presented in this dataset derive from the routine genotyping with GeneSeek Genomic Profiler (GGP) Bovine 33 k SNP chip performed by ANABIC.

**Table 1:** Composition of the three analysed datasets.

Dataset	SNP chip	N. Subset SNPs	Breed	N. subjects	N. cytogenetically tested	N. SNP-based diagnosis
1	GGP Bovine 33 k	75	Maremmana (MRM)	29	7 het ,1 hom, 22 wild type	7 het ,1 hom, 22 wild type
			Romagnola (RMG)	29	10 het, 19 wild type	10 het, 19 wild type
			Chianina (CHI)	25	3 het, 22 wild type	3 het, 22 wild type
			Podolica (POD)	34	14 het, 20 wild type	14 het, 20 wild type
			Marchigiana (MAR)	34	13 het, 21 wild type	13 het, 21 wild type
2	Illumina 50 k	75	Agerolese (AGER)	22	0	22 wild type
			Barà_Pustertaler (Barà_Pur)	24	0	24 wild type
			Burlina (BUR)	24	0	24 wild type
			Cabannina (CAB)	22	0	1 het, 23 wild type
			Calvana (CALV)	24	0	24 wild type
			Charolais (CHAR)	25	0	25 wild type
			Chianina (CHIAN)	23	0	23 wild type
			Cinisara (CIN)	30	0	13 het, 17 wild type
			Garfagnina (GARF)	23	0	3 het, 20 wild type
			Italian Brown (Bruna_Ita)	32	0	32 wild type
			Italian Holstein (Frisona_Ita)	32	0	32 wild type
			Italian Simmental (Pezz_Rossa)	32	0	32 wild type
			Limousine (LIM)	20	0	1 het, 19 wild type
			Marchigiana (MARCH)	22	0	5 het, 17 wild type
			Maremmana (MRM)	24	0	24 wild type
			Modenese (MDN)	23	0	1 het, 22 wild type
			Modicana (MOD)	29	0	1 het, 28 wild type
			Mucco Pisano (MPIS)	23	0	23 wild type
			Piedmontese (PMT)	21	0	21 wild type
			Pinzgau (PINZG)	24	0	24 wild type
			Podolica (POD)	24	0	3 het, 21 wild type
			Pontremolese (PONTR)	24	0	24 wild type
			Pezzata Rossa d'Oropa (PROropa)	23	0	23 wild type
			Pustertaler (PUST)	24	0	24 wild type
			Reggiana (REG)	26	0	26 wild type
			Rendena (REND)	24	0	24 wild type
			Romagnola (RMG)	21	0	21 wild type
			Rossa Siciliana (ROS_Sic)	24	0	5 het, 19 wild type
			Sardo-Modicana (SAM)	28	0	3 het, 25 wild type
			Sarda (SAR)	30	0	30 wild type
			Sardo-Bruna (Sardo-Bruna)	10	0	1 het, 9 wild type
			Ottonese-Varzese (VAR-OTT)	43	0	43 wild type
3	Illumina 50 k + Affymetrix 65 k	59	Dataset 2	See Dataset 2	See Dataset 2	See Dataset 2
			+Maremmana (MRM)	+ 4	1 het, 1 hom, 2 wild type	1 het,1 hom, 2 wild type
			+Romagnola (RMG)	+ 4	2 het, 2 wild type	2 het, 2 wild type
			+Chianina (CHI)	+ 2	2 wild type	2 wild type
			+Podolica (POD)	+ 4	2 het, 2 wild type	2 het, 2 wild type
			+Marchigiana (MAR)	+ 3	1 het, 2 wild type	1 het, 2 wild type
			+Cinisara (CIN)	+ 4	2 het, 2 wild type	2 het, 2 wild type

After the quality control, five individuals were excluded and a total of 153 animals (105 wild type, 47 het, and 1 hom) and 29,798 SNPs were retained.

**Figure 1:**
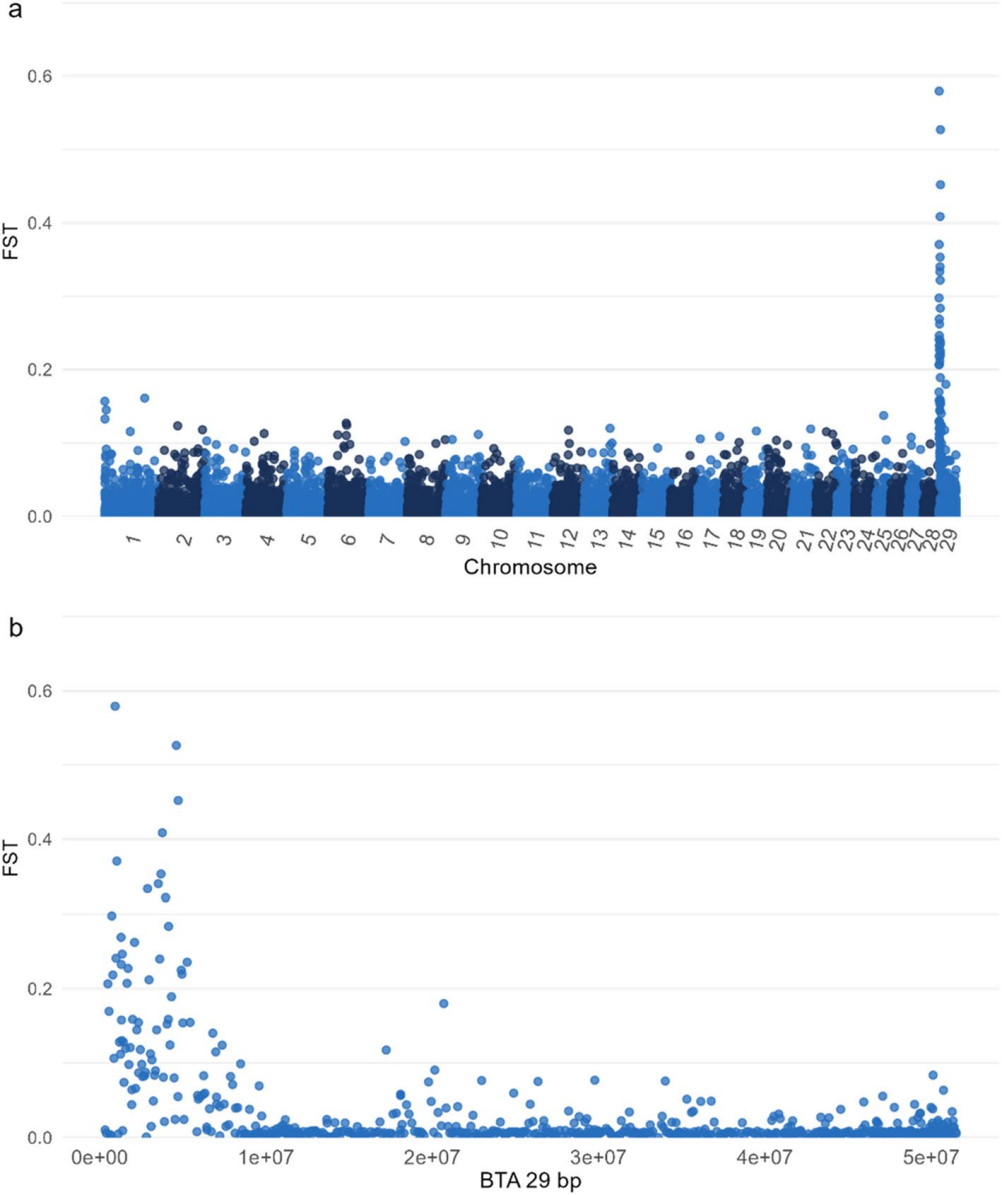
Manhattan plots representing the FST values obtained from the comparison between subjects karyologically diagnosed as rob(1;29) wild type vs. carriers. (**a**) all chromosomes; (**b**) chromosome 29.

Using this first dataset, we performed the FST analysis comparing wild type and subjects with the translocation of all the breeds, clearly identifying a peak on BTA29 (Fig. 1a). On the other hand, no signals were detected on BTA1. A further examination of chromosome 29 highlighted that the differentiating region spanned the first 6 Mb of BTA29 (Fig. 1b). This genomic region coincides with the one previously identified^14^.

**Figure 2:**
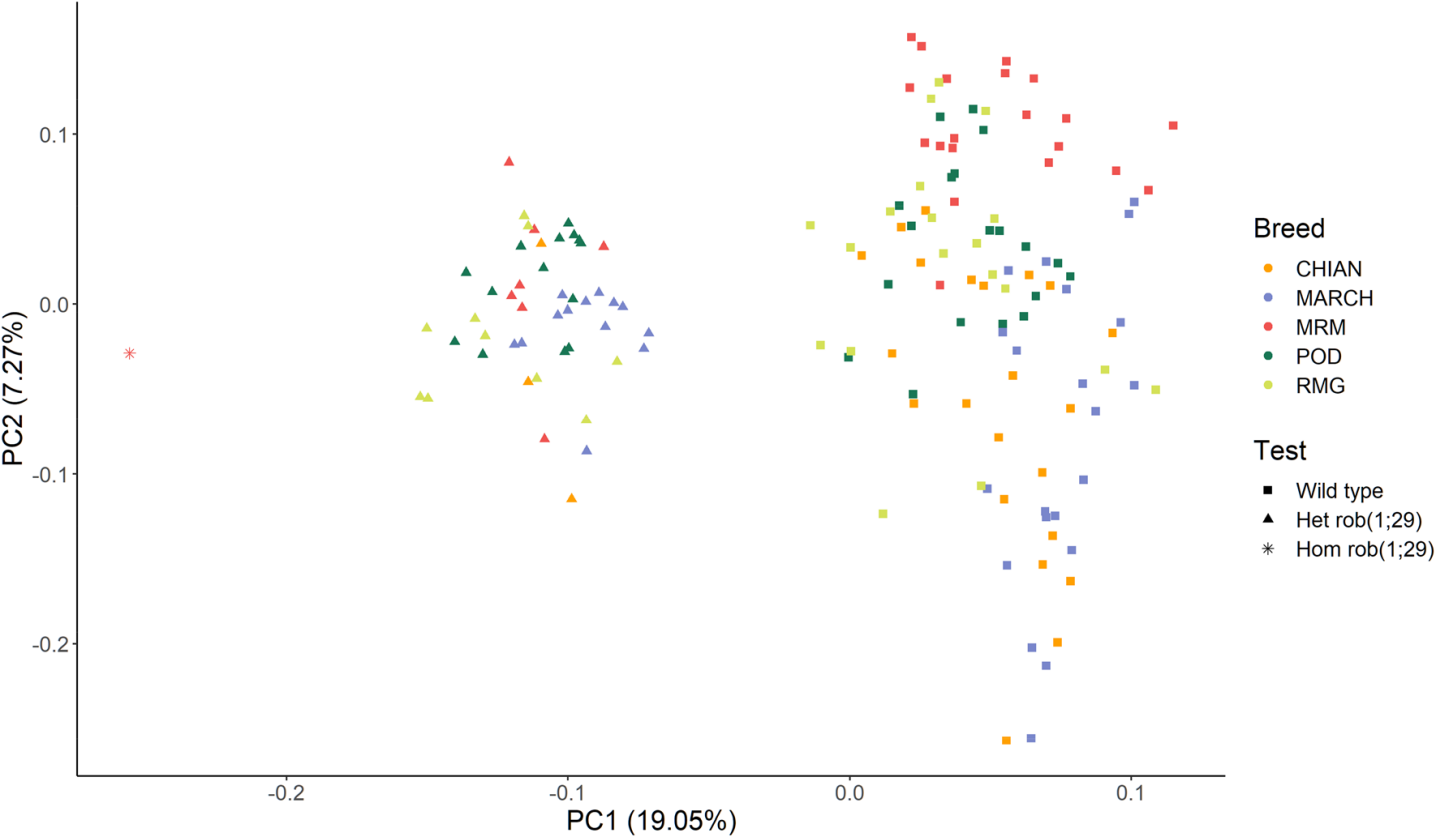
Principal component analysis (PCA) of the SNPs within the first 6 Mb of BTA29 for Dataset 1.

In light of the above, we decided to compare this region in the animals of the dataset using a principal component analysis (PCA). Specifically, only the 75 SNPs that were correctly identified by the genotyping analysis for the homozygous Maremmana individual were included. The three groups, i.e., wild type, het, and hom, were evidently distinguishable, with hom and wild type subjects at opposite ends along the first principal component and het subjects halfway between the two (Fig. 2).

To confirm the three groups observed in the PCA results we also extracted the haplotypes using the alleles of the hom individual as reference.

All the rob(1;29) carriers had at least 71 SNPs (or 99% of the correctly genotyped SNPs) that were identical to those of the hom subject. On the other hand, all the wild type subjects had no more than 64 alleles (85%) in common. The haplotype concordance for all the subjects of Dataset 1 is reported in Table S1. A discriminant test confirmed that a subject presenting at least 71 SNPs or 98.7% of genotyped SNPs in common with the hom subject’s haplotype are classified as rob(1;29) carrier with 100% specificity and 100% sensibility.

### Dataset 2 and 3

In order to investigate if this method could be applied to other breeds genotyped with a different SNP chip we used publicly available data coming from the BOVITA project, encompassing 800 subjects belonging to 32 Italian breeds and genotyped using the Illumina 50 k SNP chip. After the quality check, no individuals were excluded and 38,902 SNPs were retained. A total of 75 SNPs were comprised in the first 6 Mb of chromosome 29 and thus were used for the PCA and haplotype analyses.

**Figure 3:**
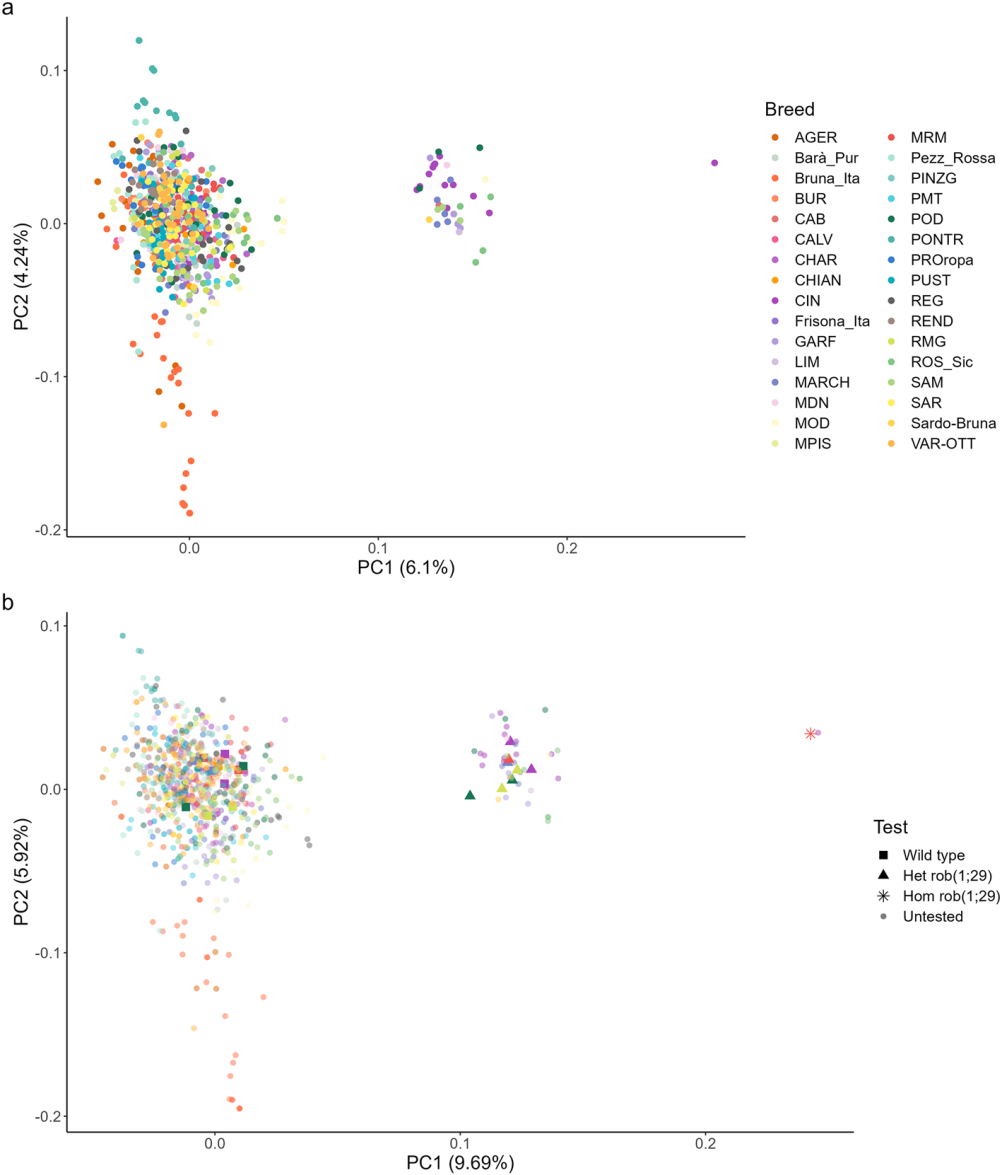
Principal component analysis (PCA) of the SNPs within the first 6 Mb of BTA29 for Dataset 2 (**a**) and 3 (**b**). For visualization purposes, untested subjects (shared between both datasets) have been plotted with increased transparency.

Again, we could observe that the subjects clearly constituted three well-defined groups, which also indicated the presence of a hom individual belonging to the Cinisara breed (Fig. 3a).

In particular, Table 1 reports the proportion of subjects presenting the translocation for each breed as inferred from our analyses. Notably, we found carriers of the translocation not only in breeds well-known for presenting it, such as Podolica (12.5%) and Marchigiana (22.7%), but also in six breeds that are not routinely screened for this problem, namely, Cinisara (43.3%, including one homozygous carrier), Garfagnina (13.0%), Italian Limousine (5.0%), Modenese (4.4%), Cabannina (4.17%), and Modicana (3.45%). Given that Cinisara breed was highly represented among the subjects with the translocation, we performed an additional sampling of 24 individuals of this breed to carry out cytogenetic analyses. We confirmed the high prevalence of rob(1;29) translocation in this breed, with 58% of subjects that were diagnosed as het. These findings hold various implications for future studies and the management of this particular breed. Notably, to the best of our knowledge, only one other cattle breed, the Portuguese Barrosã, exhibits a higher prevalence of this translocation^10,15^, which makes the Cinisara an exceptionally valuable case study to investigate the effects on fertility of this translocation and its potential role as a reservoir for homozygous individuals. On the other hand, given the potential risks associated with reduced fertility in a breed that already has a small population size, it is advisable to implement a screening and mating program for rob(1;29) in the Cinisara breed. Such a program would help mitigate the negative consequences of this translocation and safeguard the genetic diversity and fertility of this population.

A final control on this dataset was performed by adding 21 karyologically tested subjects, both heterozygous and wild type, belonging to the 5 ANABIC breeds (including the homozygous Maremmana individual) as well as to the Cinisara breed. This additional genotyping was performed using the new Affimetrix 65 k SNP chip, which shares more than 40,000 SNPs with Illumina 50 k chip. For this reason, we only retained the SNPs in common between the two chips, for a total of 32,201 SNPs and 821 individuals. This allowed us to obtain the Dataset 3.

The analyses previously described for Dataset 1, i.e., the PCA analysis and the discriminant analysis on the haplotypes, were applied to this dataset using the 59 SNPs that resulted correctly genotyped by Affymetrix 65 k chip in the hom Maremmana individual.

In the PCA analysis, the subjects maintained their previous subdivision and the tested individuals clustered within the expected groups. Interestingly, we observed a perfect overlap between the tested hom Maremmana and the Cinisara that these analyses identified as hom (Fig. 3b).

The evaluation of the haplotypes confirmed the perfect correspondence between the two hom animals and also showed that all the subjects declared as carrier by either the PCA analysis or the cytological test had at least 55 SNPs (98% of the correctly genotyped SNPs) in common with the hom Maremmana, whereas the wild type subjects had no more than 50 SNPs (85%) in common (Table S1). Thus, the discriminant analysis suggests that a number of SNPs of at least 55 or a percentage of correctly genotyped SNPs above 98.3% is enough to correctly identify rob(1;29) individuals with 100% specificity and sensibility.

The discovery of a nearly identical haplotype among carrier subjects from various Italian cattle breeds of different origins is highly significant. This finding lends support to the hypothesis that a shared ancient common ancestor may have played a crucial role in the dissemination of rob(1;29) across all cattle populations^14^. Future research investigating non-Italian breeds may further corroborate this hypothesis.

## Conclusions

In this study, we analysed the regions involved in cattle Robertsonian translocation 1;29 (rob(1;29)) using SNP data routinely generated and analyzed by breed associations. The currently employed cytogenetic screening for this translocation have limitations such as high costs, specialized personnel requirements, the need for fresh blood samples, and prolonged waiting times for diagnosis, which hinder comprehensive screening efforts. These limitations often result in analyses being limited in females, potentially perpetuating the translocation to their offspring. Our results show that the use of SNP data represents a practical and cost-effective approach that overcomes these drawbacks, enabling widespread pre-screening and prompt identification of carriers.

Provided that a reference dataset is available, the analysis of principal components and haplotypes in the first six Mb of BTA29 proved to be highly effective in distinguishing rob(1;29) homozygous and heterozygous carriers with 100% sensitivity and specificity. This region, indeed, is known to be subjected to an inversion during rob(1;29)^14^. Significantly, all the rob(1;29) carriers exhibited the same haplotype as the homozygous carrier subjects we analysed. This discovery strongly supports the theory of a common ancestor responsible for the widespread dissemination of this translocation across the entire cattle population^14^. To make screening more feasible and accessible, we recommend adding SNPs from this region to create compact, multispecies SNP chip panels. For instance, the Bovine ISAG SNP Parentage Panel, widely used for verifying cattle parentage, currently lacks SNPs within the first 6 Mb of BTA29.

SNP data analysis has broad applicability, as it potentially allows rob(1;29) screening for any genotyped animal of any breed without incurring additional costs for breed associations. Indeed, this would allow associations that already conduct screening programs to extend the evaluation to females and to biological samples other than fresh blood. Moreover, this versatile analysis could be used to screen new cattle breeds, which proves especially beneficial for smaller populations. Indeed, while validation in other breeds is advisable, it is reasonable to argue that this approach is likely to be effective across all breeds, considering the consistent nature of rob(1;29) observed in all populations studied thus far. Although this method cannot currently replace cytogenetic analyses, which have the capability to identify various chromosomal aberrations beyond rob(1;29)^16,17^, it serves as a valuable pre-screening tool applicable to a larger segment of the population.

The evaluation of SNPs allowed the detection of rob(1;29) in several Italian cattle breeds, both those known for harboring this translocation and those that had not been previously tested. Among the latter, the Cinisara, a local Sicilian dual-purpose breed of Podolian origin^18^ presented a high prevalence of rob(1;29), reaching nearly 50%, and one homozygous carrier was also identified. These findings emphasize the breed's significance as a promising candidate for initiating a screening and mating program and also highlight its value as a valuable resource for in-depth studies on effects of this translocation in cattle. Furthermore, this approach’s ability to easily identify homozygous carriers, a group that is currently underrepresented and critical for genomic research, especially in the context of whole-genome sequencing, offers a valuable resource for advancing our understanding of the underlying mechanisms and implications of this significant translocation in cattle.

In conclusion, we believe that routine implementation of our method will prove invaluable in mitigating the impact of rob(1;29) in cattle populations. Timely and accurate identification of carriers will aid in research as well as breeding management and contribute to the preservation of genetic diversity in small populations.

## Methods

### Datasets

**Figure 4:**
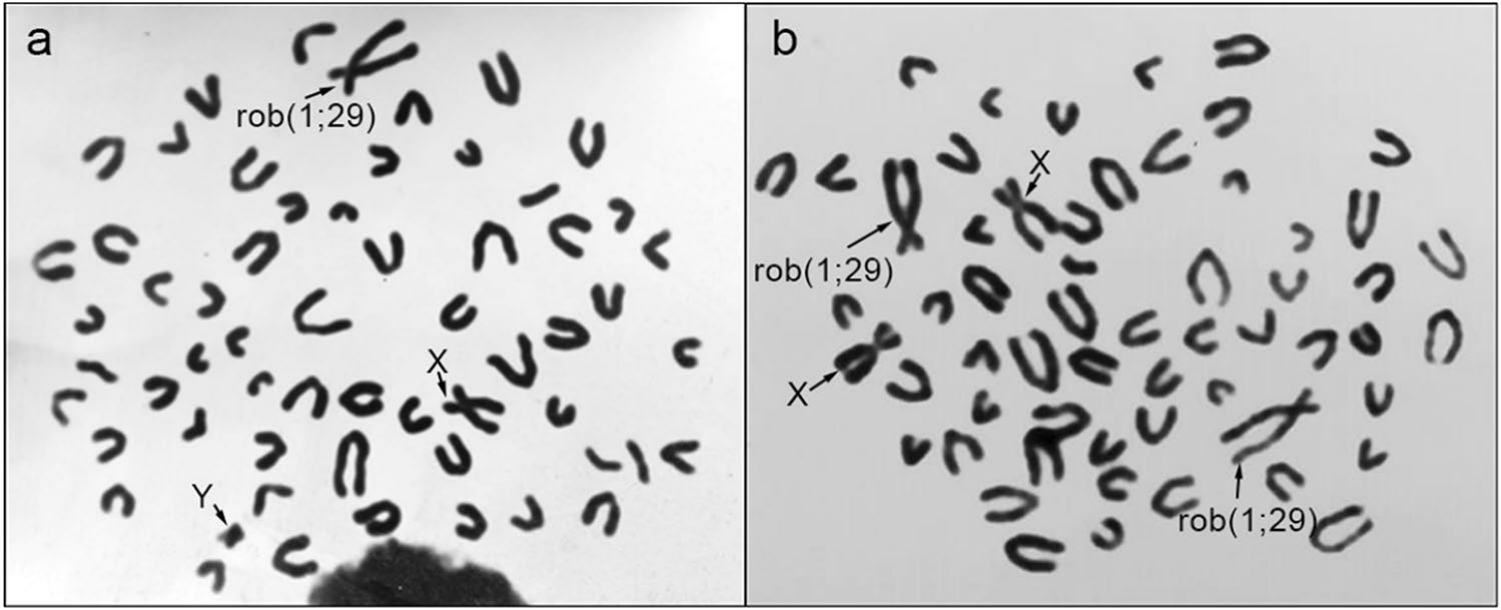
Metaphase obtained from a male heterozygous carrier of rob(1;29) translocation (**a**) and of a female homozygous carrier of rob(1;29) translocation (**b**).

For this study, three different datasets were utilized. Dataset 1 consisted of 151 subjects from 5 different breeds of Italian beef cattle managed by ANABIC: Maremmana, Podolica, Chianina, Romagnola, and Marchigiana. All these subjects had previously undergone routinely cytogenetic testing for the rob(1;29) (Fig. 4) and genotyping using the GeneSeek Genomic Profiler (GGP) Bovine 33 k SNP chip. Thus, all the data of this dataset derived from routine analyses and were provided by ANABIC association.

Dataset 2 corresponded to the publicly available version of the BOVITA project dataset, which comprised 800 subjects from 32 Italian bovine breeds genotyped using the Illumina 50 k SNP chip.

Additionally, 21 cytologically tested subjects from the five ANABIC breeds and the Cinisara breed were added to this dataset. These ANABIC subjects were in common with Dataset 1, whereas four Cinisara subjects derived from an additional sampling aimed at verify the high prevalence of rob(1;29) emerged from our analyses. Specifically, a total of 24 not directly related Cinisara individuals were randomly selected. Blood samples were collected and stored in EDTA (for genotyping) and sodium heparin (for karyologic analysis) collection tubes. This sampling was carried out according to the Ethics Committee’s statement of the University of Messina number 046/2020.

Cell cultures were established following the standard methodology^19^, and metaphases were stained with Giemsa. The genotyping of the newly added subjects was performed using the Affymetrix 65 k SNP chip, which shared a substantial number of SNPs with the Illumina 50 k SNP chip. This allowed us to create Dataset 3, consisting of a total of 821 subjects and the SNPs in common between the Affymetrix 65 k and Dataset 2.

### Data filtering and subset extraction

All three datasets were filtered only for genotyping quality of the single subjects using the PLINK parameter -geno set to 0.05^20^.

Subsequently, a subset of SNPs within the first 6 Mb of chromosome 29 and correctly genotyped in the rob(1;29) homozygous Maremmana subject (present in all the three datasets) was extracted. This specific region was selected according to previous literature and further confirmed through an FST analysis conducted between wild type subjects and those presenting the translocation using the PLINK software^20^ on the first dataset.

### Principal component analysis and haplotype concordance

Principal Component Analysis (PCA) was performed on the subset of SNPs within each dataset using PLINK^20^.

The results were then compared with the concordance of the haplotypes of each individual with that of the homozygous Maremmana subject. The extraction of haplotypes was carried out using an in-house script.

### Canonical discriminant analysis

Using JMP 16 software, a canonical discriminant analysis was conducted on the SNPs belonging to the haplotypes for datasets 1 and 3. The aim was to identify the smallest possible number of SNPs capable of distinguishing subjects with rob(1;29) translocation from wild type ones.

### Institutional review board statement

Cinisara additional sampling was approved by the Ethics Committee of the University of Messina (protocol code number 046/2020).

The investigation of the remaining data does not involve “animal experiment” as defined by the exemptions outlined in the Italian legislative decree n. 26/2014 (Dir. 2010/63/UE on the protection of animals used for scientific purposes).

### Supplementary Information

Supplementary Table S1.

## Data Availability

Dataset 2 consisted of BOVITA project publicly available data deposited at https://osf.io/vh72y/?view_only=8f9b5fc86ffa4835adf4bb2df1543ab8 (accessed on 1st May 2023). Dataset 1 and additional samples of Dataset 3 may be available upon request to the corresponding author (A.B.).
